# Treated Follicular Lymphoma, Recurrent Invasive Pneumococcal Disease, Nonresponsiveness to Vaccination, and a Unique Pneumococcus

**DOI:** 10.1155/2012/386372

**Published:** 2012-12-30

**Authors:** Clare Murphy, Donald Inverarity, Claire McGoldrick, Lindsay Mitchell, Pamela Paterson, Louise Thom, Giles Edwards

**Affiliations:** ^1^Department of Infectious Disease, Monklands Hospital, Monkscourt Avenue, Airdrie ML6 0JS, UK; ^2^Department of Medical Microbiology, Monklands Hospital, Airdrie ML6 0JS, UK; ^3^Department of Clinical Haematology, Monklands Hospital, Airdrie ML6 0JS, UK; ^4^Scottish Haemophilus, Legionella, Meningococcus and Pneumococcus Reference Laboratory (SHLMPRL), House on the Hill, Stobhill Hospital, 133 Balornock Road, Glasgow G21 3UW, UK

## Abstract

A nonneutropenic patient with treated low-grade non-Hodgkin's (Follicular) lymphoma and secondary hypogammaglobulinemia recovered from pneumococcal pneumonia and septicemia (serotype 7F; ST191) subsequent to influenza A H1N1 (2009). Both infections were potentially vaccine preventable. The patient then developed pneumococcal meningitis due to a serotype 35F pneumococcus with a unique Multilocus Sequence Type (ST7004) which was not vaccine preventable. Patient management was influenced by host predisposition to pneumococcal infection, antibiotic intolerance, and poor response to polysaccharide pneumococcal vaccine. Indirect immunofluorescence with anti-human immunoglobulin confirmed a poor or intermediate response to Pneumovax II. Prophylactic erythromycin was initiated, and immunoglobulin transfusions were also commenced as a preventive strategy. ST7004 is a single locus variant of ST1635 which has been associated with the serotype 35F capsule in England. The *spi* gene in ST7004, which differentiates it from ST1635, is the same as the *spi* gene present in ST191 which could have arisen from the first disease episode suggesting that horizontal gene transfer may have occurred between different populations of pneumococci present within the patient in an attempt to evade vaccination selection pressure.

## 1. Introduction

We explore the difficulties managing recurrent, life threatening, invasive pneumococcal (IPD) disease in an immunocompromised patient with treated Follicular lymphoma and antibiotic intolerance. Host factors therefore both predisposed to IPD and unresponsiveness to polysaccharide pneumococcal vaccine and hampered the optimal antibiotic treatment of the patient. We describe a novel strategy of prophylaxis against IPD utilizing regular immunoglobulin transfusions and long-term oral antibiotic prophylaxis with erythromycin which since its implementation has prevented further episodes of IPD. Interestingly we also discovered that the most recent of the patient's episodes of IPD was due to a unique sequence-type of *Streptococcus pneumoniae* with evidence to suggest that it may have been generated *in vivo* in this patient between episodes of IPD by horizontal gene transfer [1] both creating a new sequence type and facilitating IPD due to vaccine escape by generation of a nonvaccine-included serotype [2]. 

## 2. Case Presentation

A 76-year-old Caucasian lady with a past medical history of non-Hodgkin's (Follicular) lymphoma, secondary hypogammaglobulinemia, bronchiectasis with recurrent lower respiratory tract infections, and aortic stenosis presented acutely unwell, but not neutropenic, with one week of fever and productive cough. On the day of admission she had been found unresponsive. 

On admission she had a Glasgow Coma Scale of 10/15, with eyes opening to speech. She was pyrexial (40.3°C) and tachycardic with a blood pressure of 148/91. An ejection systolic murmur was audible along with bibasal coarse crepitations. 

Finger prick testing revealed hypoglycaemia which was treated with 50% dextrose with an increase in the blood glucose but no improvement in the patient's general condition. Electrocardiograph and chest X-ray did not show any acute abnormalities. Echocardiograms were performed on days 2 and 13 with no vegetations or evidence of infective endocarditis.

The patient underwent a computed tomography head scan on day one which was reported as being normal. A lumbar puncture was performed which obtained a visibly turbid cerebrospinal fluid (CSF) with a white cell count of 7600 cells per mm^3^ (95% neutrophils), a red cell count of 250 cells per mm^3^, undetectable glucose level, and a protein level of 7.02 g/L. Gram stain of CSF revealed Gram-positive diplococci. Unfortunately there was no growth after 48 hours incubation of CSF, but blood cultures grew *S. pneumoniae* (serotype 35F, ST7004) susceptible to penicillin (minimum inhibitory concentration = 0.012 mg/L), erythromycin (MIC = 0.06 mg/L), vancomycin (MIC < 1 mg/L), and ceftriaxone (MIC < 0.06 mg/L). Polymerase chain reaction of the CSF using a real-time multiplex assay to identify the* lytA* gene confirmed the presence of pneumococci in CSF, and further PCR for the 7 Multilocus Sequence Typing (MLST) house keeping genes identified this also as an entirely new sequence type (ST7004) currently unique to this patient.

The patient was known to develop a rash when given penicillin and so was initially treated with intravenous ceftriaxone 2 g twice daily and vancomycin 750 mg twice daily. Dexamethasone 2.5 mg four times daily was added orally on day 2 for a 4 day course. On day 6 treatment was rationalised to ceftriaxone 2 g twice daily alone on which the patient remained until discharge on day 15 having made a full recovery with no neurological deficit.

Eight months previously this patient had required admission for sepsis while recovering from Influenza A H1N1 (2009) and was found to have left basal pneumococcal pneumonia with septicaemia due to a vaccine preventable serotype (serotype 7F, ST191) treated initially with clarithromycin then levofloxacin. Prior to both episodes of invasive pneumococcal disease (IPD) she had received Pneumovax II (Sanofi Pasteur). We assessed her antibody response to the pneumococcal serotypes present in Pneumovax II using indirect immunofluorescence with anti-human immunoglobulin which indicated a poor response to many serotypes including 7F (Table 1) in keeping with her known secondary hypogammaglobulinemia.

Given the failure to respond to vaccination as a likely consequence of hypogammaglobulinemia and the predisposition this has given to recurrent IPD this patient now receives transfusions of 20 g normal human immunoglobulin (Kiovig, Baxter) every 3 weeks to prevent further reinfection. Prophylactic daily erythromycin has been commenced, and this patient is offered yearly seasonal influenza vaccination. 

## 3. Discussion

Infection with *S. pneumoniae* can manifest as pneumonia, meningitis, and septicemia. These can be aggressive and life threatening even in an immunocompetent host. Hypogammaglobulinemia as a consequence of treated Follicular lymphoma is recognized and predisposes to bacterial infection such as recurrent IPD [3] as does the chronic lung disease of bronchiectasis as well as priming due to recent infection with influenza A [4]. Failure to respond to pneumococcal polysaccharide vaccination in the context of secondary hypogammaglobulinemia is also well known [3]. Penicillin allergy considerably limits the antibiotic treatment options for manifestations of IPD. Consequently not only did this patient have multiple factors predisposing her to IPD, but she also had multiple factors which hampered their treatment and prevention, a potentially fatal combination.

Having successfully treated two episodes of IPD within 9 months, we devised a novel prophylaxis regimen based on regular administration of intravenous immunoglobulin to provide passive immunity [5] and daily prophylactic erythromycin. The patient continues to be offered seasonal influenza vaccination annually. No further episodes of IPD have occurred since commencing these. 

**Figure 1:**
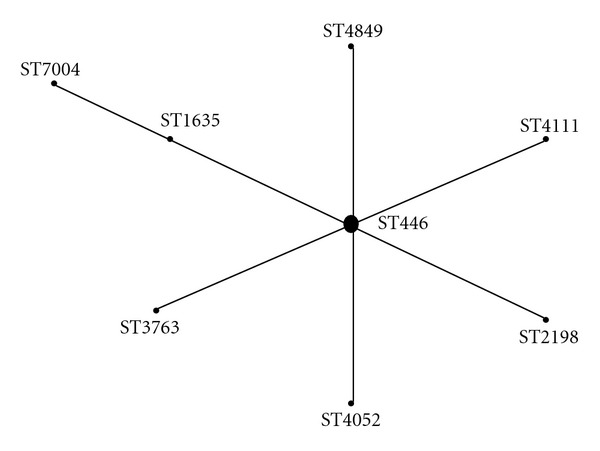
An e-BURST (electronic-Based Upon Related Sequence Types) diagram which illustrates that ST7004 is a single-locus variant related to ST1635 as part of a clonal complex with ST446 as its founder. Interestingly in the MLST database, ST446 is always associated with a serotype 35F capsule.

Additionally, the unique predisposing factors in this patient may have generated an environment that facilitated the generation of a unique *S. pneumoniae in vivo*. In Scotland all CSF and blood isolates of *S. pneumoniae* undergo serotyping and MLST.  Table 2 illustrates that the pneumococcus causing the episode of meningitis is comprised of a new unique sequence type (ST7004) which is closely related to another pneumococcal sequence type identified in the United Kingdom (ST1635) also with a serotype 35F capsule.  Figure 1 also shows a relationship between ST7004 and ST1635 which are single locus variants of each other belonging to a clonal complex with ST446 as its founder which according to the MLST database has always been found expressing a serotype 35F capsule. The *spi* gene that distinguishes them could plausibly have arisen through horizontal gene transfer [1] from the ST191 pneumococcus that caused the initial episode of septicaemia which may have persisted from several months earlier.

These related sequence types from the same patient suggest that an environment where horizontal gene transfer of the *spi* gene between the ST191 (presumably persisting from the first episode of IPD) and possibly a serotype 35F-associated ST1635 may have been generated to create an entirely new sequence type, ST7004, with a serotype 35F capsule which would indicate that possibly capsular switching had also occurred [6]. It is unusual to be able to see evidence of such potential horizontal gene transfer and capsular switching to occur *in vivo* in the same patient. As this patient had been vaccinated against *S. pneumoniae*, albeit with a suboptimal response, there may have been enough selection pressure produced to drive serotype replacement [2] in the pneumococcal population colonizing this patient without enough host immunity to prevent infection.

## 4. Conclusions

This case illustrates the difficulties of managing recurrent IPD when there are significant host factors such as hypogammaglobulinemia and immunocompromise due to haematological malignancy, penicillin allergy, bronchiectasis, and recurrent lower respiratory tract infections. Such factors predispose both to pneumococcal infection and failure to respond to vaccination. The case also suggests genetic changes in the pneumococcal population which enabled it to persist and cause recurrent infection by means of generating a unique genotype.

## Figures and Tables

**Table 1:** Patient's response to Pneumovax II determined by indirect immunofluorescence with anti-human immunoglobulin. Poor response indicates a level below that believed to be protective and intermediateandindicates a response that is likely to be protective but low in healthy volunteers.

Nature of host response	Serotypes tested
Poor	1, 2, 5, 6B, **7F**, 9V, 12F, 15B, 17F, 18C, 19F, 22F, 23F, and 33F
Intermediate	3, 4, 8, 9N, 10A, 11A, 14, 19A, and 20

**Table 2:** Comparison of sequence types demonstrating great similarity between serotype 35F-associated ST7004 (a new ST) and ST1635 which is a serotype 35F-associated ST which has previously been seen in the United Kingdom.

MLST	*aroe *	*gdh *	*gki *	*recP *	**spi**	*xpt *	*ddl *
ST191 (serotype 7F)	8	9	2	1	**6**	1	17
ST7004 (serotype 35F)	10	7	4	19	**6**	40	27
ST1635 (serotype 35F)	10	7	4	19	**10**	40	27
